# Singular Case of Erysipelas

**Published:** 1863-10

**Authors:** W. H. Byford

**Affiliations:** Professor, &c., Chicago Medical College


ARTICLE XXVII.

### SINGULAR CASE OF ERYSIPELAS.

BY W. H. BYFORD, A.M., M.D., Professor, &c., Chicago Medical College.

The subject of the attack, in this case, was Mr. B., a German, 45 years of age, he is strong and plethoric. He lives in Cincinnati, but was on a visit to his friends in this place. When at home, he occupies a large, well-ventilated house, and lives well, including an habitual, but not intemperate, (in the German sense of the word,) supply of wine and malt liquors. While here, which was but a week or ten days before he was attacked, he occupied a small room with low ceiling in a badly-ventilated house, with two children, his wife, and a servant. In a dietetic point of view, he fared as at home. He is in the habit, for the last six or eight years, of having a moderate attack of erysipelas once a year. I mention the above facts that they may have due weight in accounting for the singular course pursued by the disease.

He was attacked, June 5th, 1863, with a chill, succeeded by a moderate grade of febrile reaction, which was accompanied with a blush of redness on the middle of the nose. This redness developed into an erysipelatous form of inflammation, sufficiently painful to confine the patient to bed for five or six days. On the 12th, seven days from the beginning, the inflammation had invested the whole head, and the advancing margins met on the occipital region. The skin upon the upper part of the face and forehead had vesicated and was undergoing exfoliation. This process was about completed; the patient had become very comfortable on the 18th, and I took my leave flattering myself that convalescence was almost complete.

Next day, in the afternoon, I was again sent for to see my patient, when I found that a chill and fever, the evening before, had been followed by a recommencement of inflammation on the nose, at the point where the first attack began. The erysipelatous inflammation spread over the same surface, ran its course, and subsided on the 24th. It was not attended with so much distress or fever as the first, but there were febrile reaction, pain, and tumefaction, and the creeping advance of inflammation of erysipelas. When the whole head was surrounded the second time, the inflammation and symptoms subsided, so that, on the 25th, I again took leave of the case, thinking now I was quit of the singular circumstance.

To my astonishment, however, I was called upon to witness a repetition of the same phenomena, which commenced on the morning of the 26th and finished upon the 3d day of July. Thus, my patient had experienced three perfectly developed erysipelatous inflammatory attacks; the inflammation travelling over an extensive surface in a perfect manner each time, in the course of one month, and succeeded each other so closely that they might be considered relapses of the same attack.

The treatment was began, in the first attack, by ordering four grains of calomel at bedtime, to be followed, in the morning, by citrate of magnesia. Soon as it had operated, the patient was to have twenty drops of the muriated tincture of iron in some water every four hours. He was to have cloths kept constantly wet with apple vinegar on the inflamed part. This treatment was followed steadily for the whole of the first attack; and the tincture was left off only when I ceased my attendance. It was resumed and continued during both of the subsequent attacks, up to the 2d of July. At this time, although there was still some inflammation, I sent him out in the air, and bade him live out door as much as possible, and change his lodgings to a larger and more commodious room. From this time he was convalescent and experienced no further illness, except the annoyance of three or four abscesses under different parts of the scalp, left behind as the effects of the erysipelas. The tincture of iron, acid washes to the inflamed surface, attention to the bowels, and liberal diet constituted the treatment. I have been induced to publish this short sketch of my case from two considerations:—

1st.—In a practice of twenty-five years, during which time I have seen my share of erysipelas, I have, in no instance, known a relapse of erysipelas, that is, an immediate recurrence and prevalence of the inflammation on and over the same surface even once, let alone twice, as in this case; and I have no recollection of reading of an instance of the kind, although I confess not to have examined the subject with special reference to this circumstance; and,

2d.—Because the recurrence took place at a time when the patient might be supposed to be saturated with acid and iron, generally and locally.

I prefer making no comments, leaving my readers to draw their own inferences.

